# Quantum confinement and coherent transport in ultrathin ${\mathbf {{{Bi}_{2}}}\mathbf {{Se}_{3}}}$ nanoribbons

**DOI:** 10.1038/s41598-025-23622-7

**Published:** 2025-10-31

**Authors:** Kiryl Niherysh, Xavier Palermo, Ananthu P. Surendran, Alexei Kalaboukhov, Raitis Sondors, Jana Andzane, Donats Erts, Thilo Bauch, Floriana Lombardi

**Affiliations:** 1 Quantum Device Physics Laboratory, Department of Microtechnology and Nanoscience, Chalmers University of Technology, 41296 Göteborg, Sweden; 2 Institute of Chemical Physics, Faculty of Science and Technology, University of Latvia, 1586 Riga, Latvia

**Keywords:** Topological materials, Magnetotransport, Quantum interference effects, Materials for devices, Nanoscale materials, Nanoscale devices, Nanoscale materials

## Abstract

In recent years much progress has been made in realizing topological insulator (TI) nanostructures where the reduced dimensions should help to diminish the contributions from bulk carriers and enhance quantum confinement. Though nm thick 3D-TI nanoribbons exhibiting topological properties are still difficult to reproducibly synthesize. Here we demonstrate the growth of ultrathin ${\mathbf {{{Bi}_{2}}}\mathbf {{Se}_{3}}}$ nanoribbons by a simple catalyst-free physical-vapour deposition, where the tuning of the material evaporation time plays a crucial role in determining the ultimate thickness of the nanoribbons. Magnetotransport and Hall effect measurements show that at thicknesses close to 10 nm the transport features are affected by Altshuler-Aronov-Spivak like coherent orbits at low magnetic fields, while Shubnikov-de Haas oscillations take over at high fields. The observed phenomena originate from the topological surface states and dominate the nanoribbon transport. Ultrathin nanoribbons also show pronounced conductance oscillations as a function of gate voltage, that can be attributed to ballistic transport and quantized sub-bands. The results highlight the importance of material growth to exploit the unique properties of topological surface states, establishing 3D-TI nanoribbons as a promising platform for a variety of novel applications.

**Supplementary Information:**

The online version contains supplementary material available at 10.1038/s41598-025-23622-7.

## Introduction

The properties of the topologically protected Dirac surface states in 3D topological insulator (3D TI) nanostructures are promising for a variety of applications including quantum computing^1–4^, spintronics^5–8^, thermoelectric devices^9–12^, and realization of single-electron charge pumps with high accuracy for metrology^13^. However, the contribution from the trivial bulk charge carriers often masks the exotic properties of the topological surface states. To take full advantage of the protected surface states, one needs to tune the chemical potential inside the band gap and close to the Dirac point. One effective way to eliminate the bulk contribution is chemical compensation doping of nanoribbons^14–16^; however, this procedure significantly reduces the mobility of surface Dirac electrons^17,18^. An alternative approach to reduce the bulk contribution maintaining high mobility is to increase the surface-to-volume ratio by growing 3D-TI materials with reduced dimension such as nanoribbons^19,20^.

In our previous work we demonstrated the growth of ${\mathbf {{{Bi}_{2}}}\mathbf {{Se}_{3}}}$ nanoribbons using our standard catalyst-free physical-vapour deposition (PVD) method^21^. However, in this study, we had a lower yield of nanoribbons with a thickness of less than 15 nm. Here we show that by properly tuning the deposition conditions of our PVD technique, we can identify a regime of growth that allows to obtain stoichiometric ${\mathbf {{{Bi}_{2}}}\mathbf {{Se}_{3}}}$ nanoribbons with thicknesses below 15 nm and lengths up to 5–10 ${\mathrm{\upmu m}}$. For the thinnest nanoribbons, the growth mechanism changes from a layered to rough regime, varying the morphology of nanostructures, which affects the transport properties of the topological surface states of nanoribbons. In addition to the quantum oscillations in a magnetic field clearly observed in nanoribbons thicker than 15 nm, our combined magnetoresistance and Hall effect measurements reveal a distinct transport regime for thinner ribbons (below 15 nm), where Altshuler-Aronov-Spivak (AAS)-like orbits dominate the transport at low magnetic fields, while Shubnikov-de Haas (SdH) oscillations can still be observed at high fields. Moreover, we demonstrate a clear signature of quantized energy band structure in the form of an oscillatory behaviour of the longitudinal resistance as a function of back-gate voltage in our thinnest (12 nm thick) and narrowest (below 100 nm in width) nanoribbons. Overall, our results highlight the importance of material growth and geometrical confinement to be able to use the unique properties of topological surface states for devices beyond the state of the art.

## Experimental results and discussion

### Synthesis of ultrathin ${\mathbf {{{Bi}_{2}}}\mathbf {{Se}_{3}}}$ nanoribbons

**Fig. 1:**
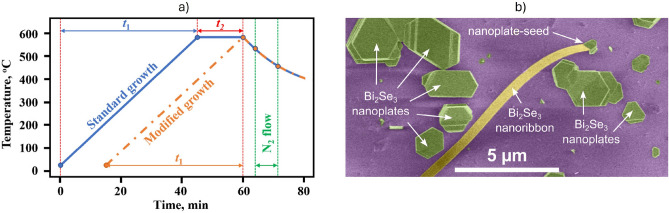
(**a**) Schematic of the synthesis process. The solid blue line corresponds to the standard growth described in *Ref.*^21^, and the orange dot-dashed line corresponds to a modified growth with t_2_ = 0 min. (**b**) False-color SEM image of a ${\mathbf {{{Bi}_{2}}}\mathbf {{Se}_{3}}}$ nanoribbon and the nanoplate seed.

${\mathbf {{{Bi}_{2}}}\mathbf {{Se}_{3}}}$ nanoribbons were synthesized via catalyst-free physical vapour deposition in a GSL-1100X tube furnace (MTI Corporation). ${\mathbf {{{Bi}_{2}}}\mathbf {{Se}_{3}}}$ powder was used as a source material, and placed in the center of the furnace tube where the temperature reaches 585 °C during the synthesis. A glass substrate (25$\times$75 mm) was placed downstream from the source material. Before the synthesis, the tube was first flushed with N_2_ gas for 5 min to create an inert atmosphere. The scheme of the synthesis process is shown in Fig. 1a.

First, the furnace was heated up from room temperature to 585 °C in a time interval t_1_ = 45 min. The substrate temperature at this moment was from 430 to 230 °C at its “*hot*” and “*cold*” ends, respectively. After that, the furnace was kept at the temperature of 585 °C for a time t_2_ that was varied between 15 (standard growth^21^) and 0 min (modified growth, these work), and afterwards the furnace was turned off to cool down naturally. When the temperature in the furnace center decreased to 540 °C, an N_2_ gas flow was introduced in the tube with a dynamic pressure of 26 Torr, initiating the growth of the nanoribbons. The flow was terminated once temperature reached 475 °C, and the tube was filled with N_2_ to atmospheric pressure.

During the standard growth process^21^, nanoplates are deposited on the substrate for a time t_1_+t_2_. They serve as seeds for the subsequent formation of nanoribbons during the N_2_ streaming. As the top surface of the nanoplate is chemically saturated by selenium atoms^22^, adatoms adsorbed there from the gas phase cannot form covalent bonds. Thus, they diffuse in the direction of the gas flow and bond to the nanoplate crystal at the edges, which leads to a much faster growth rate in the N_2_ flow direction, forming long crystalline nanoribbons^21^. Since the cross-section of the nanoribbons is correlated to that of the seed nanoplates from which they grow, as shown in Fig. 1b, it can be influenced by changing the conditions in which the nanoplates are grown. The amount of deposited material is proportional to the partial pressure of the evaporated source and evaporation time^23^, and then the thickness of nanoribbons can be changed by adjusting only these parameters. In this work, the deposition time t_2_ was reduced from 15 to 0 min, while the pressures p_1_ and p_2_ - measured when the heater is switched on and when it reaches the set temperature of 585 °C, respectively - were kept constant.

**Fig. 2:**
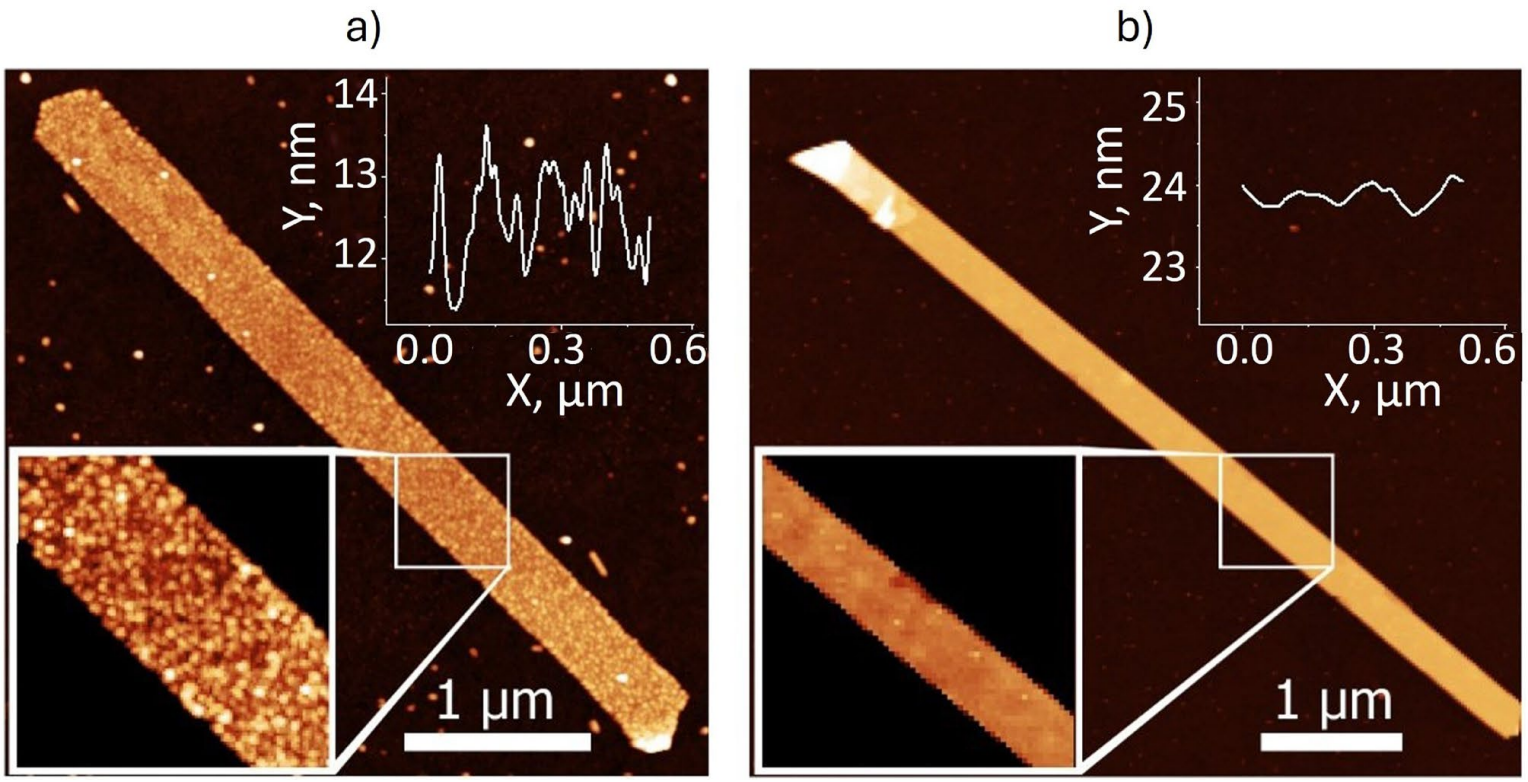
(**a**) AFM image of a 12 nm thick nanoribbon grown with the rough mechanism. The inset is the surface profile with RMS roughness around $\sim 670~$pm. (**b**) AFM image of a 22 nm thick nanoribbon in which the rough-to-smooth growth transition mechanism occurred. The inset is the surface profile with RMS roughness around $\sim 130~$pm. The *y*-axis range is identical to the one of the inset in panel (**a**) to emphasize the significant difference.

The synthesis parameters not only influence the geometry of the nanoribbons, but also determine their growth mechanism, affecting their transport properties^24^. Fig. 2a and b show atomic force microscopy (AFM) images and surface profiles for 12 and 22 nm thick nanoribbons, respectively. The thin nanoribbon (Fig. 2a) shows the presence of small grains on the surface, while the thicker one (Fig. 2b) has an atomically flat surface. The average surface roughness for the thin nanoribbon is $\sim 0.67~$nm, while for the thicker ribbon this parameter is $\sim 0.13~$ nm. These images seem to indicate that the growth kinetics of thin nanoribbons differ from that of thick ones.

Here we propose a possible growth scenario that can occur when time t_2_ is changed. During the vapour-solid (VS) process, the formation of nanoribbon/nanowire-like nanostructures is determined by growth kinetics^25,26^. As mentioned earlier, the top surface of the nanoplates (seeds) is chemically saturated. As a result, newly arriving growth species (adatoms) will diffuse to side edges with atomic steps, ledges, and kinks^27,28^. Previously, it was shown that the two-dimensional nucleation probability on the surface of nanoribbons can be described as^25–27^:

1 $$\begin{aligned} P_N = B \exp {\left( -\frac{\pi \sigma ^2}{k^2T^2\ln {\left( p/p_0\right) }}\right) } \end{aligned}$$

where $P_N$ is the nucleation probability, *B* is a constant, $\sigma$ is the surface energy, *k* is the Boltzmann constant, *T* is the absolute temperature, $p$ is the actual vapour pressure, and $p_0$ is the equilibrium vapour pressure corresponding to temperature *T*. If the vapour pressure $p<p_0$, the chemical potential of the crystal is larger than that of the vapour and the crystal should sublimate. However, if $p>p_0$ the vapour should crystallize. In our experiment, the deposition of nanostructures proceeds in the supersaturated state $(p/p_0 )>1$. Therefore, the supersaturation ratio $(p/p_0 )$ and temperature are two dominant processing factors in controlling the morphology of the products in the VS growth process^27^.

The amount of evaporated material at the start of nanoribbons growth in the case of t_2_ = 0 min is much less than in the case of the standard growth, where t_2_ = 15 min. Nanoplates (seeds) deposited only over time t_1_ have smaller thicknesses in comparison with flakes deposited during time t_1_+t_2_. However their side edges with atomically rough steps, regardless of the plate thickness, act as effective “*catalyst*” that initiates the growth of nanoribbons in the lateral direction. As in the case of the vapour-liquid-solid growth mechanism, the edge side area of the “*catalyst*” plate, from which the nanoribbon growth occurs, can be saturated with adatoms more easily for thinner seeds compared to thicker ones^29^. The value of supersaturation at the edge surface tends to decrease for plates with higher thicknesses. Previously, it was also shown that a decrease in supersaturation may lead to the rough-to-smooth transition of the crystal growth mechanism^30–32^. We have experimentally found that during the same deposition process, nanoribbons less than 15 nm thick have a rough surface, while ribbons more than 15 nm thick have a smooth morphology, indicating a 2D growth mechanism. This suggests that a transition from rough to smooth growth takes place in our experiment. Moreover, this growth scenario is consistent with the appearance of a high yield of thin nanoribbons. At some point, the growth particles from the vapour cannot saturate the side edge of very thick seeds, and the chemical potential of the crystal itself becomes larger than that of the vapour $(p_0>p)$. In this case, the growth of the thickest nanoribbons may stop, while the growth rate of crystals from saturated seeds increases with a decrease in their thickness^29^. As we will show below, the difference in morphology between thicker and thinner nanoribbons strongly affects the transport properties of the topological surface states.

### Transport measurements of ${\mathbf {{{Bi}_{2}}}\mathbf {{Se}_{3}}}$ nanoribbons

**Fig. 3:**
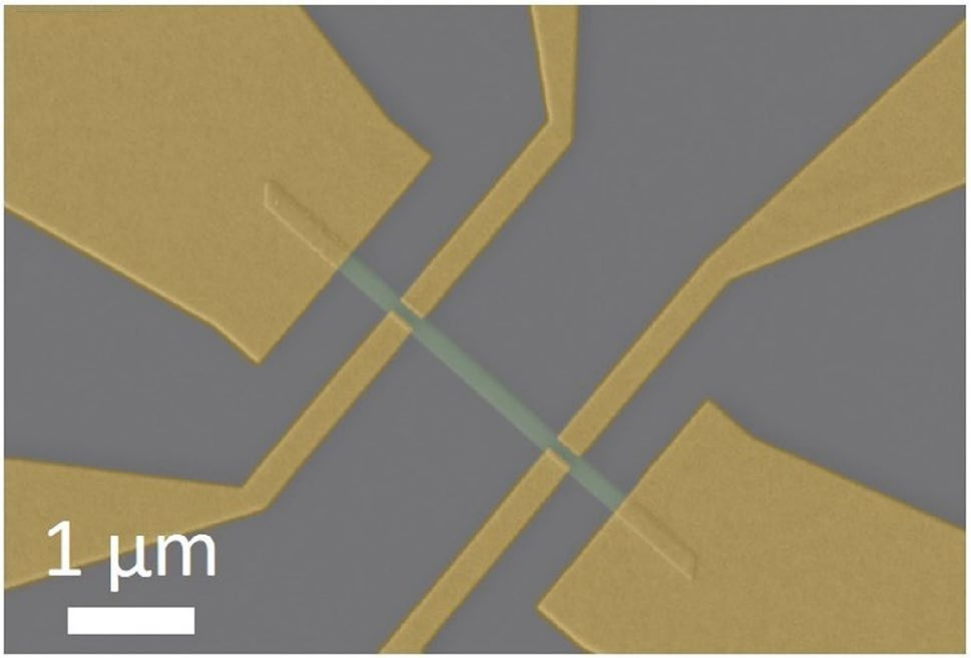
False-color SEM image of a fabricated device based on a ${\mathbf {{{Bi}_{2}}}\mathbf {{Se}_{3}}}$ nanoribbon (green) transferred to a Si/SiO_2_ substrate (grey). The metal electrodes are shown in yellow.

${\mathbf {{{Bi}_{2}}}\mathbf {{Se}_{3}}}$ nanoribbons synthesized using the parameter t_2_ = 0 min were transferred to a Si/SiO_2_ (300 nm) substrate for transport measurements. After the transfer, several nanoribbons were selected using optical and atomic force microscopies. The electrical leads were patterned via electron-beam lithography to create contacts for magnetoresistance and Hall effect measurements. Ar^+^-ion beam etching was used to remove the native oxide from the top surface of the nanoribbons prior to the deposition of Ti/Au contacts (3/80 nm) (Fig. 3).

**Fig. 4:**
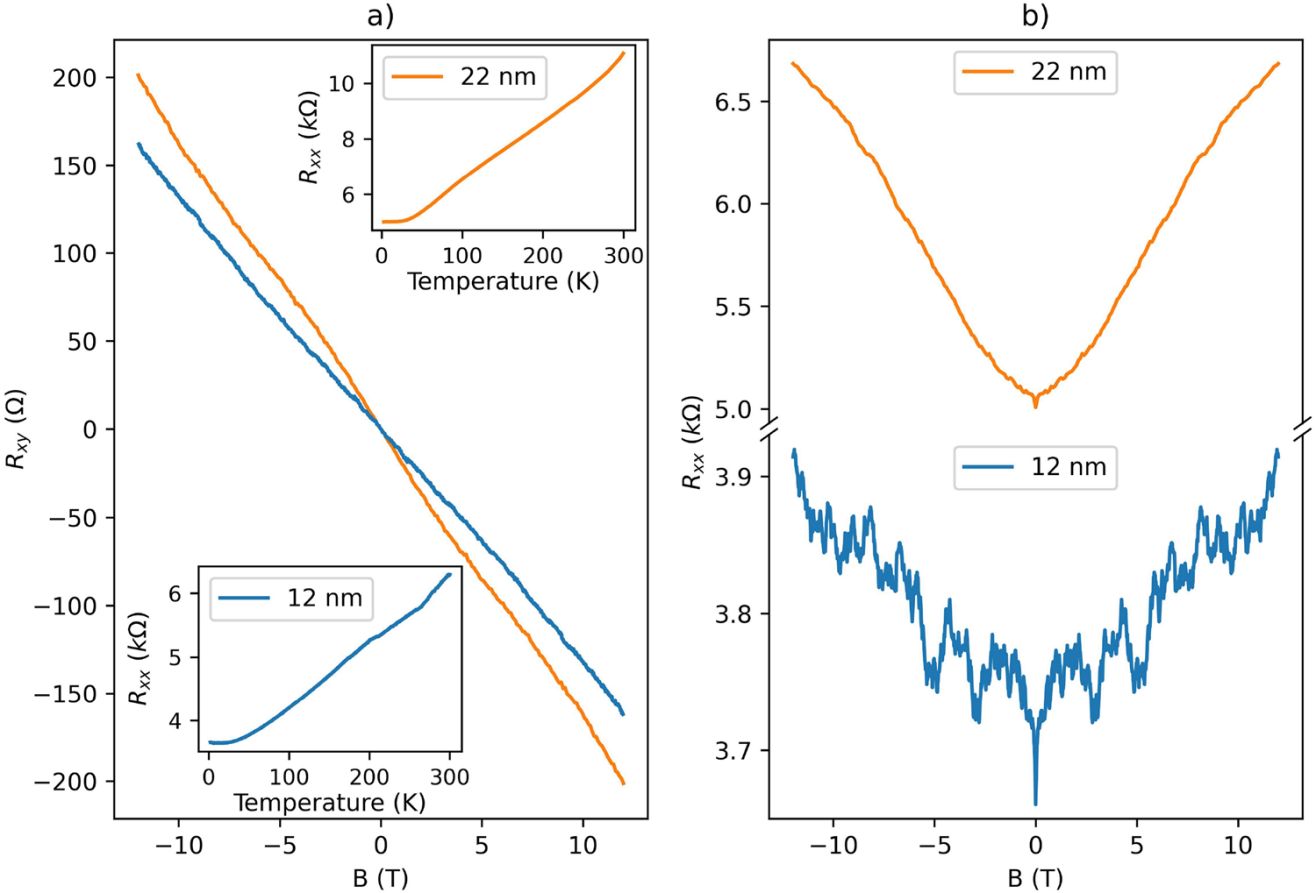
The magnetic field dependence of the (**a**) transverse $R_{xy}$ and (**b**) longitudinal $R_{xx}$ resistances for 12 nm (blue, *Device I*) and 22 nm (orange, *Device II*) thick ribbons measured at 2 K, respectively. The insets in (**a**) represent the temperature dependence of $R_{xx}$ for the two devices.

The longitudinal resistance $R_{xx}$ was measured from room temperature down to 2 K (shown in the insets of Fig. 4a). The metallic behavior is observed for both thin and thick ribbons as well as for previously reported ${\mathbf {{{Bi}_{2}}}\mathbf {{Se}_{3}}}$ nanoribbons/nanowires with different thicknesses^21,33^. This dependence is expected since the ${\mathbf {{{Bi}_{2}}}\mathbf {{Se}_{3}}}$ nanostructures grown by the PVD method usually contain native defects such as selenium vacancies, which act as electron donors (*n*-type doping), thereby shifting the Fermi level into the conduction band^34,35^. We measured the Hall resistance $R_{xy}$ to estimate the sheet carrier density $n_{2D}$ of individual nanoribbons (Fig. 4a). The latter is given by:

2 $$\begin{aligned} \frac{1}{n_{2D} \cdot e} = \frac{dR_{xy}}{dB} \times g, \end{aligned}$$

where *e* is the elementary charge, and *g* is geometrical correction factor. Due to overlapping of the nanoribbon with contact electrodes (the non-ideal Hall bar geometry, see Fig. 3), the measured Hall voltage needs to be corrected^36^. Using a finite element simulation method (COMSOL Multiphysics), we numerically solved the current continuity equation and obtained the correction factor $g\approx$ 4 for our Hall bar geometries and contact resistances^19^. The values of $n_{2D}$ extracted from the data are respectively 1.2 × 10^13^ cm^−2^ for *Device I* (thickness *t* = 12 nm, and width *w* = 360 nm), and 8.8 × 10^12^ cm^−2^ for *Device II* (thickness *t* = 22 nm, and width *w* = 310 nm), while the negative slope indicates *n*-type carriers for both devices. The estimated values of Hall mobility obtained for *Device I* and *Device II* are $\mu _1$ = 1075 and $\mu _2$ = 1210 cm^2^/V·s, respectively.

The magnetoresistance $R_{xx}$ as a function of a magnetic field (up to 12 T) applied perpendicularly to the surface of the nanoribbons was also measured at low temperatures (2 K). Thin ${\mathbf {{{Bi}_{2}}}\mathbf {{Se}_{3}}}$ nanoribbons with thickness below 15 nm show well-pronounced magnetoresistance oscillations in the entire range of the magnetic fields, while for thicker nanoribbons they are only visible at high fields (Fig. 4b). As we will discuss below, this difference can be explained by the coherent scattering of electron waves from surface irregularities associated with the peculiar morphology of the thin nanoribbons (due to different growth mechanisms compared to the thicker ones), which leads to the appearance of AAS-like orbits.

**Fig. 5:**
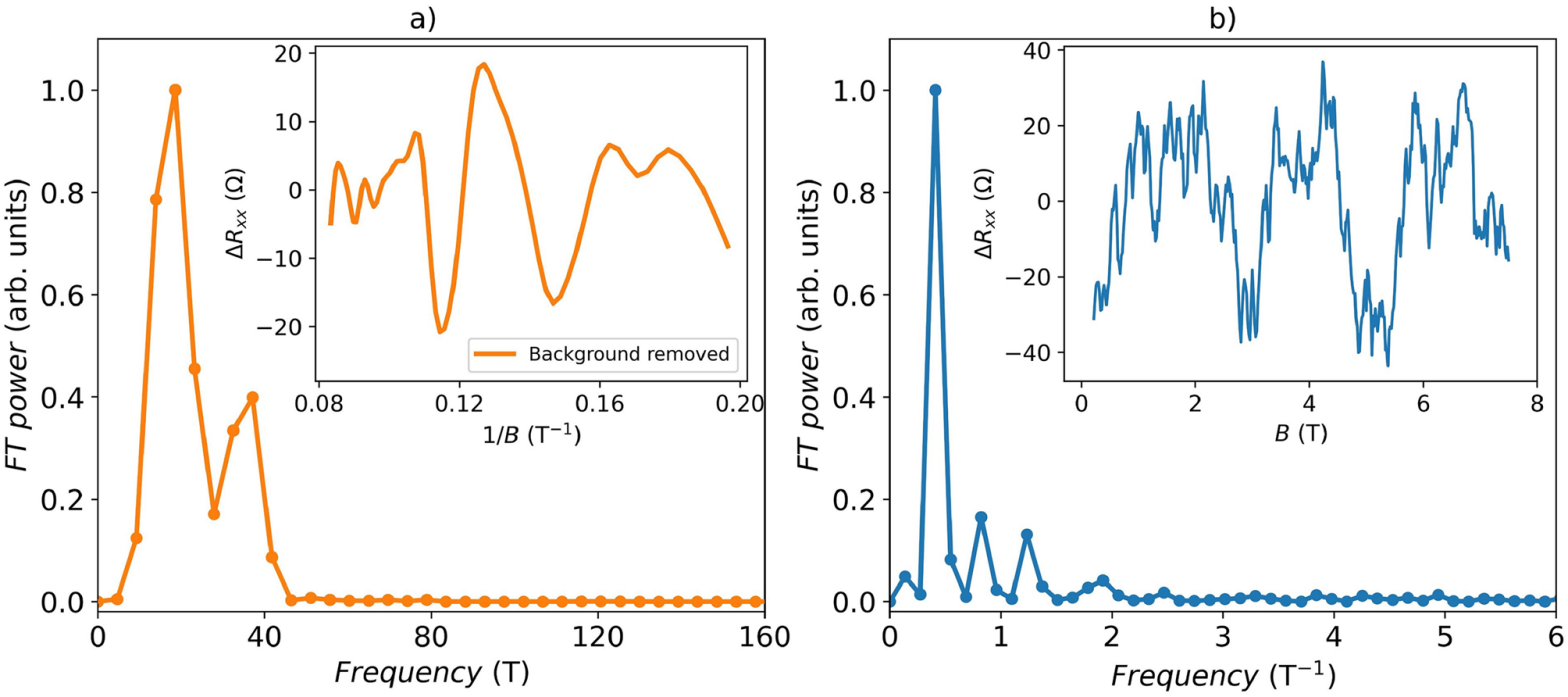
Power spectra of (**a**) $\Delta R_{xx}(1/B)$ (shown in the inset) for a 22 nm thick (*Device II*) nanoribbon and (**b**) $\Delta R_{xx}\left( B\right)$ (in the inset) for a 12 nm thick ribbon (*Device I*) measured at 2 K, respectively. A 5$^{th}$-order polynomial was subtracted to remove the magnetoresistance background.

For the thick nanoribbon (*t* = 22 nm, *Device II*), pronounced oscillations were found by subtracting a polynomial background (Supplementary Information 1, Fig. S1) and plotting the data in 1/*B* (see inset of Fig. 5a). The observed oscillations are associated with a quantum phenomenon known as Shubnikov-de Haas oscillations and can be related to the cross-sectional area of the Fermi surface in momentum-space via the Onsager relation^33,37^. Fourier transform (FT) analysis (Fig. 5a) on the SdH oscillations was performed to find out the oscillation frequencies. Thus, the frequencies $F_{1}^{II}=18.7$ T and $F_{2}^{II}=37.2$ T were identified, corresponding to $n_{2D\_1}^{II}=4.52$ $\times$ 10^11^ cm^−2^ and ${n_{2D\_2}^{II}=8.99}$ $\times$ 10^11^ cm^−2^ according to the Onsager relationship. The presence of two frequency peaks is at the origin of the observed beating pattern of the SdH oscillations^38^.

We have previously demonstrated a multi-frequency pattern similar to the one reported here for nanoribbons thicker than 30 nm^33^. Notably, the corresponding frequencies remained unaffected by applying a high back-gate voltage, indicating that they originate either from the bulk states or from the top Dirac surface states (at the interface of the nanoribbon with vacuum)^33^. This interpretation is supported by nearly one order-of-magnitude discrepancy between the 2D carrier densities extracted from Hall effect measurements and those derived from SdH oscillations. As has been shown, a charge accumulation layer with $n_{2DEG}\approx$ 1.0 $\times$ 10^13^ cm^−2^ is formed at the nanoribbon-substrate interface and dominates in the Hall conductance^19,33^. Since the bottom topological surface states (TSSs) overlap with the accumulation layer with much lower mobility, SdH oscillations due to this 2DEG do not usually appear in the magnetoresistance. Considering these findings, we conclude that the observed frequencies in the SdH spectrum originate from either bulk states or surface carriers at the interface with a vacuum, consistent with the behaviour reported in *Ref.*^33^.

As shown in Fig. 4b, thin nanoribbons (less than 15 nm) exhibit instead a strong oscillatory pattern in the entire range of a magnetic field. The phenomenology of these oscillations is compatible with that of universal conductance fluctuations (UCF). The latter represents quantum interferences of electron diffusion paths with different lengths, leading to aperiodic oscillations in magnetic field^39^. However, the FT calculated from $\Delta R_{xx}\left( B\right)$ below 7 T (to exclude the possible impact of SdH on the magnetoresistance) shows instead a dominant peak at $F_{UCF}^{I}=0.41$ T$^{-1}$ (Fig. 5b, *Device I*). A well-defined periodicity is unusual for conventional UCF (Supplementary Information 2, Fig. S2). However, it can be explained if the fluctuations originate from Altshuler-Aronov-Spivak-type orbits, with similar characteristic areas that become accessible because of the specific surface morphology of the thin nanoribbons.

A comparable phenomenology was previously reported in *Refs.*^40,41^, where periodic Aharonov-Bohm (AB) oscillations were observed in the magnetotransport of ${\mathbf {{{Bi}_{2}}}\mathbf {{Se}_{3}}}$ films and ${\mathbf {{Bi}_{2}}\mathbf {{Te}_{3}}}$ flakes. These materials exhibited characteristic pyramidal domains on their surfaces, and the length scale of the observed AB orbits corresponded to the typical perimeter of triangular terraces found on the surface. Tunneling spectroscopy performed in *Refs.*^42,43^ further demonstrated that the local density of states is strongly enhanced near these step edges on ${\mathbf {{Bi}_{2}}\mathbf {{Se}_{3}}}$ and ${\mathbf {{Bi}_{2}}\mathbf {{Te}_{3}}}$, confirming that they act as preferential electron trajectories. In our case, the characteristic length scale of the observed oscillations corresponds to the circumference of similarly sized circular grains on the surface of the thin nanoribbons (see Fig. S3, Supplementary Information 3), strongly indicating that the periodic magneto-fingerprint originates from coherent electron wave scattering at their step edges. While AB-type oscillations were dominant in the previous studies, they are unlikely to appear in our system due to the significantly larger dimensions of the device containing a large number of grains, which would average out such effects. Nonetheless, the presence of uniform circular grains on the nanoribbon surface can support closed-loop paths around their perimeter, enabling AAS-type interference (Supplementary Information 4, Fig. S4). Given the uniformity in grain dimensions, we expect a periodic modulation in conductance as a function of magnetic field, with a characteristic period determined by the enclosed area. Unlike AB oscillations, AAS oscillations are not averaged out in ensembles of rings and should persist, leading to robust interference signatures in the magnetoconductance^44^.

The characteristic AAS area of these orbits can be calculated as $S=\phi \times F_{UCF}^I$, where $\phi = h/2e$ is the magnetic flux quantum relevant for AAS oscillations. From the extracted orbit area $S=0.0008$ $\mu$m$^2$ one can estimate a characteristic path length for the electrons as $L~\sim ~$2$\sqrt{\pi S}=100$ nm. The calculated value 2$R_{AAS}=32$ nm (for circular orbits $L=2\pi R_{AAS}$) is in good agreement with the characteristic size of the surface irregularities of about 29.4±1.6 nm, which was extracted using the two-dimensional fast Fourier transform of the AFM image presented in the inset of Fig. 2a (Supplementary Information 3).

**Fig. 6:**
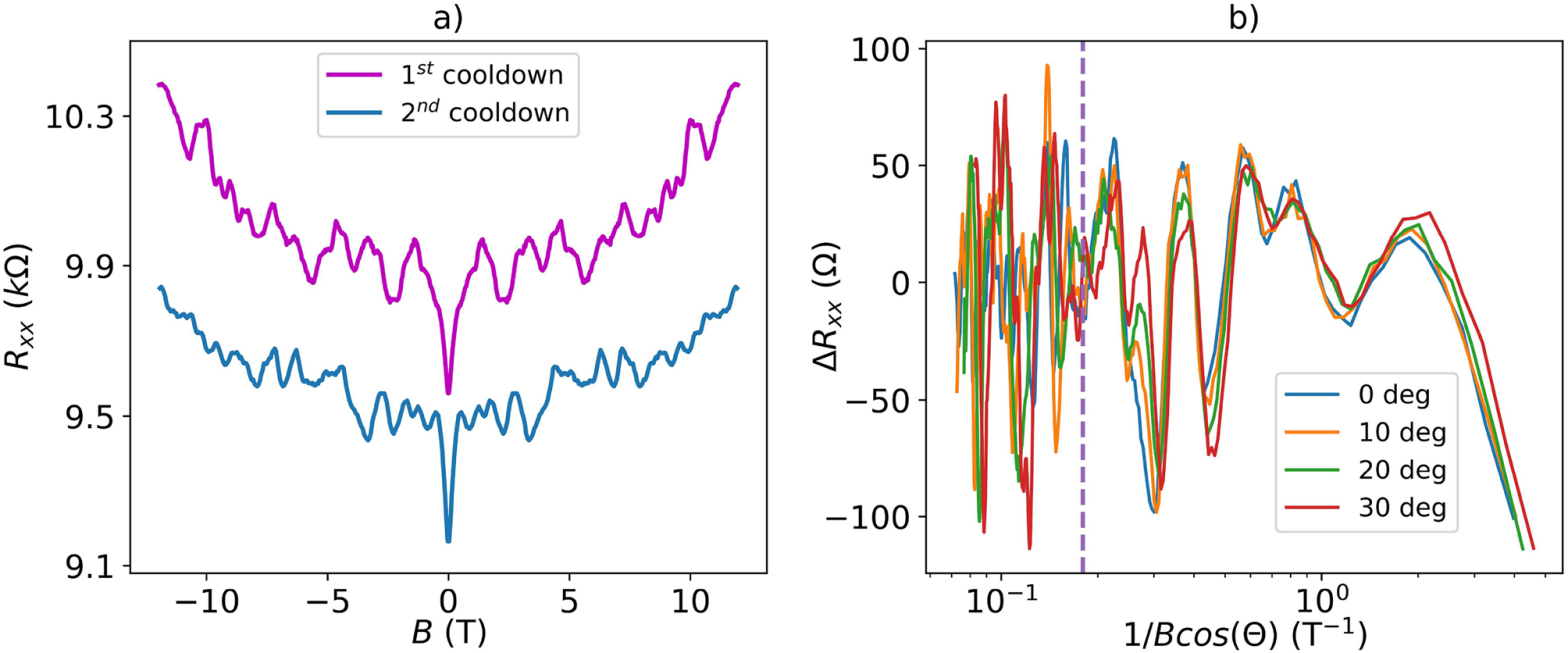
(**a**) The magnetoresistance of *Device III* (thickness *t* = 12 nm, and width *w* = 85 nm) as a function of a magnetic field after the first and second cooldowns, measured at 2 K. (**b**) Background-removed (5$^{th}$-order polynomial) resistance $\Delta R_{xx}$ as a function of $1/B\cos {\theta }$. The *x*-axis is plotted on a logarithmic scale to show a clear data superimposition at low magnetic fields. The vertical dashed line denotes a magnetic field of 5.5 T.

Further analysis will be associated with *Device III*, fabricated using a ${\mathbf {{Bi}_{2}}\mathbf {{Se}_{3}}}$ nanoribbon with a maximized surface-to-volume ratio (thickness *t* = 12 nm, and width *w* = 85 nm). For this device, the $R_{xx}\left( B\right)$ pattern changed after warming up to room temperature (Fig. 6a), which excludes that the magnetoresistance oscillations can be only attributed to SdH oscillations (Fig. S5, Supplementary Information 5). Moreover, the onset of SdH oscillations requires the condition $\mu B \gg$ 1, where $\mu$ is the electron mobility. This condition is not usually satisfied below $5-6$ T, since typical Hall mobility values in nanoribbons are between 1200−2100 cm$^2/$V·s as reported in our previous works ^19,33^. Therefore, we attribute the observed oscillations in the low magnetic field to the coherent AAS-like orbits with a similar characteristic area, analogous to *Device I* and conventional UCF.

To determine whether these oscillations come from interference effects in the bulk or from the 2D topological surface, we have studied the angular dependence of $R_{xx}$ with respect to the out-of-plane magnetic field. In an ideal 2D electron system, the interference pattern depends only on the normal component of the magnetic field $B_\perp = B\cos {\theta }$, where $\theta$ is the angle between the direction normal to the nanoribbon plane and the orientation of the magnetic field^45^. After subtracting a 5$^{th}$-order polynomial background, the resulting oscillation patterns $\Delta R_{xx}$ measured at various angles $\theta$ superimpose if plotted against $1/B_\perp$ below 5 T, which confirms the 2D origin of UCF^40^ (Fig. 6b). However, the analysis becomes more complicated at higher fields due to the coexistence of UCF, AAS and SdH oscillations. The coherence of the AAS-like orbits associated with the specific morphology of the samples is also observable at higher magnetic fields and can overlap with SdH oscillations.

**Fig. 7:**
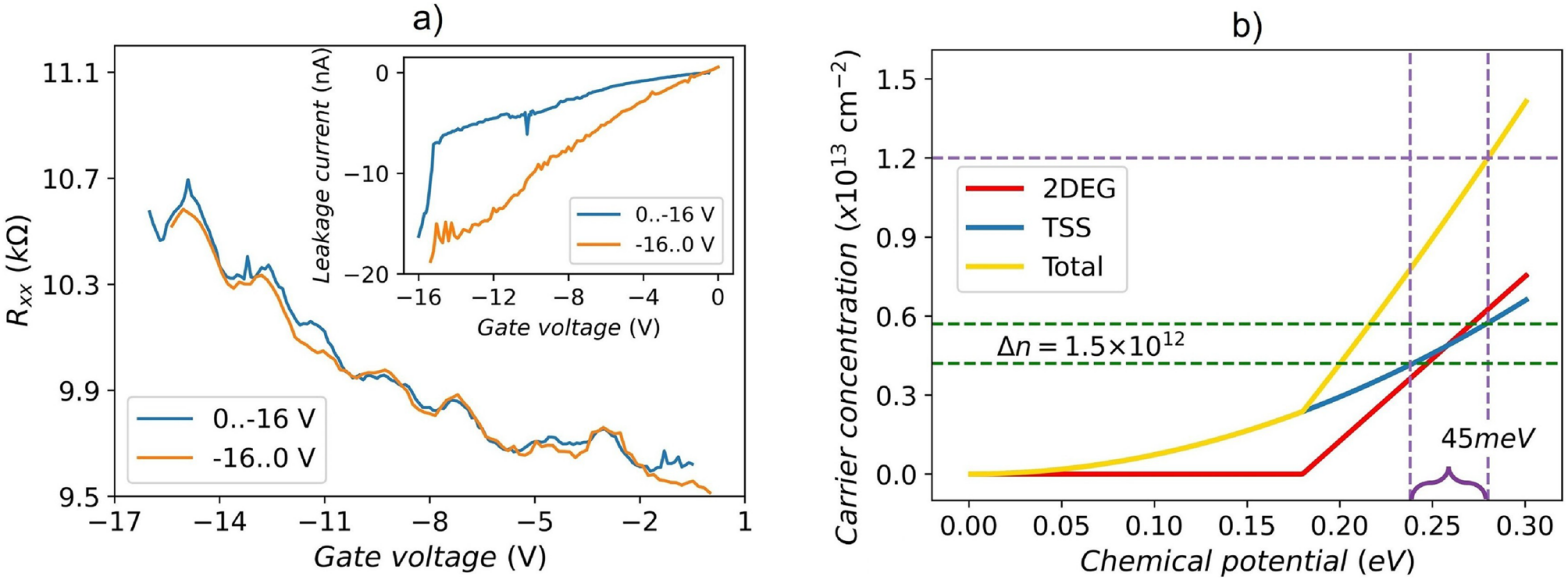
(**a**) $R_{xx}$ as a function of back-gate voltage (*Device III*, $T =$ 2 K). Blue and orange curves represent two scan sweeps with different directions (see more data in Fig. S6 of Supplementary Information 6). The leakage current was continuously monitored and kept below 3 % of the measured signal (inset). (**b**) Charge carrier concentration as a function of Fermi energy. The red line shows the dependence for a regular 2DEG, the blue line for Dirac states, and the yellow one for the substrate/nanoribbon interface, where both types of carriers exist.

The $R_{xx}$ as a function of the gate voltage has been studied for *Device III*. In addition to the expected increase of the resistance due to the depletion of the nanoribbon carriers by the gate, we also observe reproducible oscillations of $R_{xx}$ as a function of back-gate voltage (Fig. 7a). We now contend that size quantization effects are essential in our devices.

The band structure of surface carriers in TI nanoribbons is described by the momentum vector *k* along the nanoribbon axis and the angular momentum *l*^46,47^:

3 $$\begin{aligned} E_l(k) =\pm \hbar v_F \sqrt{k^2+\frac{\pi (l)^2}{S}}, \end{aligned}$$

where *l* is half-integer $\pm \frac{1}{2}$,$\pm \frac{3}{2}$,..., $\hbar$ is the reduced Planck constant, $v_F$ is Fermi velocity ($5\times 10^5$ ms$^{-1}$), *S* is the cross-sectional area of the nanoribbon. When the chemical potential crosses one sub-band, a new conduction channel becomes accessible, leading to pronounced resistance dips^46^. We argue that the oscillations observed in $R_{xx}(V_g)$ are directly related to sub-bands formation, as described by Eq. 3. To support this interpretation, we perform the following analysis: first, we calculate the carrier concentration as a function of chemical potential; second, we estimate how the carrier concentration changes with an applied gate voltage (see Fig. 7b). Finally, we compare the change in chemical potential induced by the applied gate voltage with the number of resistance oscillations observed in our measurements.

The charge carrier concentration of a trivial 2DEG is given by $n_{2DEG}={k_F^2}/{2\pi }$. The corresponding Fermi energy can be calculated as:

4 $$\begin{aligned} E_{F(2DEG)} =\frac{\hbar ^2 k_F^2}{2m^*}=\frac{\hbar ^2 \pi }{m^*}n_{2DEG}, \end{aligned}$$

where $k_F$ is Fermi wavevector, $m^*$ is effective mass, for the ${\mathbf {{Bi}_{2}}\mathbf {{Se}_{3}}}$ case $m^* = 0.15 m_e$ ^33^. For Dirac fermions the carrier concentration is $n_{SS} = {k_F^2}/{4\pi }$, and the Fermi energy can be written as:

5 $$\begin{aligned} E_{F(SS)} =\hbar k_F v_F=\hbar v_F \sqrt{4\pi n_{SS}}. \end{aligned}$$

Since the gate voltage primarily affects the charge carrier concentration at the substrate/nanoribbon interface^38^, the position of the Fermi level can be estimated from the total carrier concentration at the substrate/nanoribbon interface using Eqs. 4 and 5, considering that the conduction band minimum is located 180 meV above the Dirac point^33^. For a typical carrier concentration in our nanoribbons on a Si/SiO_2_ substrate of *n* = 1.2 $\times ~10^{13}$ cm$^{-2}$, the calculated $E_F$ is $\approx$ 270 meV. The contributions to the total carrier concentration as a function of the chemical potential measured from the Dirac point are shown in Fig. 7b: the blue line represents the bottom TSSs, the red line is the trivial 2DEG, and the yellow one is the sum of the two.

To estimate the carrier concentration change induced by the applied gate voltage, we have numerically computed the capacitance between the back-gate electrode and the bottom surface of the 3D-TI nanoribbon using COMSOL Multiphysics. So, considering a simple asymmetric metallic parallel plate geometry, where one electrode is given by the doped silicon and the other is the nanoribbon, the static surface charge carrier density per applied gate voltage $\sigma \simeq 1.5~\times 10^{-4}$ C/m^2^·V was numerically calculated.

Based on this value of $\sigma$, the change in carrier concentration at the bottom interface can be estimated using the relation $\Delta n=\sigma {V_g}/{e}$. For the maximum applied voltage ($V_g=-16$ V), this yields $\Delta n=$1.5$\times 10^{12}$ cm$^{-2}$. We can assume that the change of carrier concentration occurs mainly in the bottom TSSs (Supplementary Information 7). This is justified by the fact that the trivial 2DEG at the bottom extends well inside the bulk, and therefore does not feel the electric field of the gate potential that is screened by the TSS^38^. In this case, the $\Delta n$ corresponds to a change in the chemical potential of $\approx$ 45 meV (see Fig. 7b). For the dimensions of the nanoribbon in this study (*Device III*), we find that the distance between the sub-band minima $\Delta =hv_F/C \approx 9$ meV, where *h* is the Planck constant and $C=2(w+t)$ is the circumference of the nanoribbon with rectangular cross-section^48,49^. The change in the chemical potential would therefore correspond to the crossing of approximately 5 sub-bands which is very close to the 5 to 6 $R_{xx}(V_g)$ oscillations observed in Fig. 7a.

**Fig. 8:**
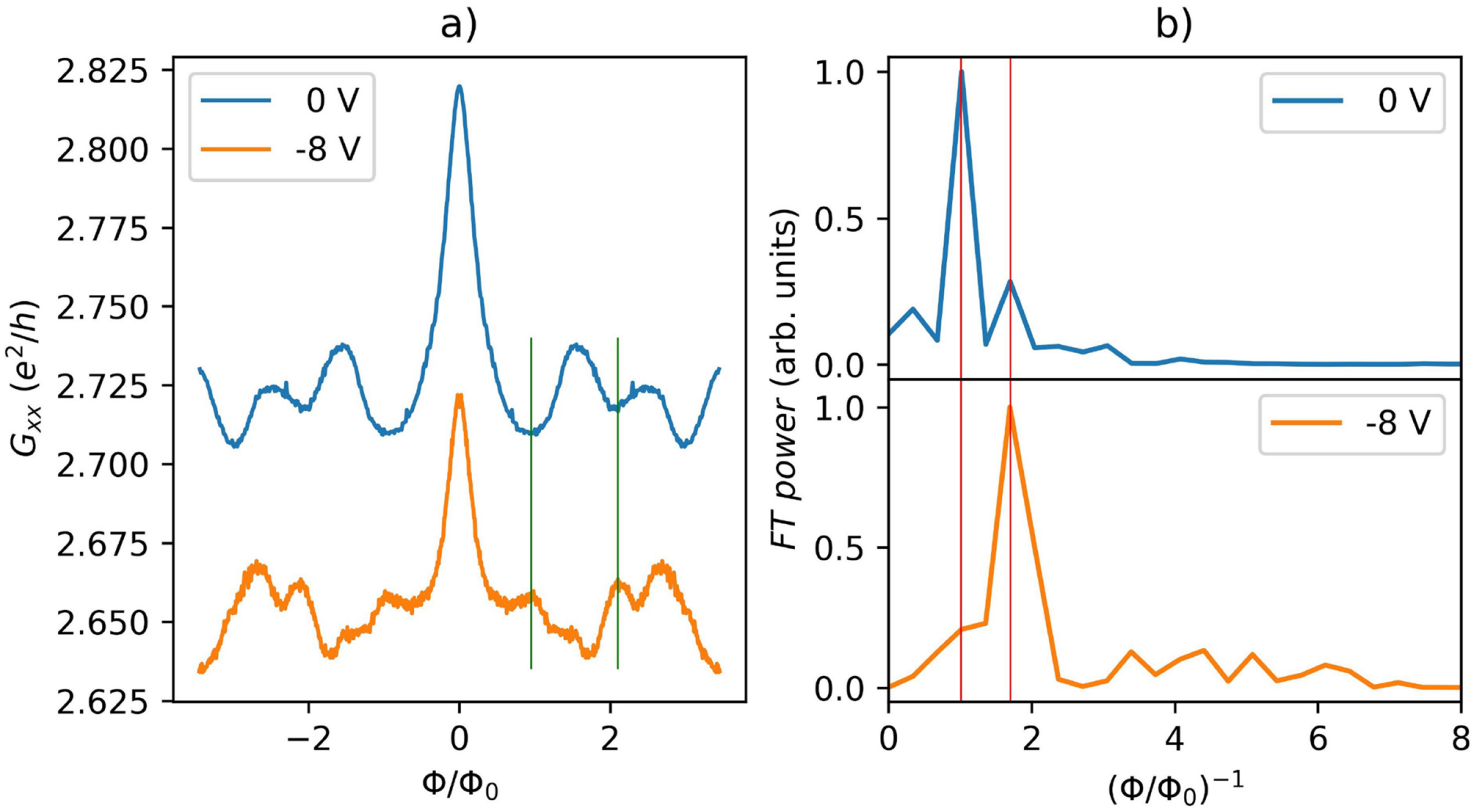
(**a**) The magnetoconductance as a function of $\phi /\phi _0$ for two different back-gate voltages 0 and $-8$ V (*Device III*). The measurements were carried out at a temperature of 2 K. (**b**) Fourier transforms of $\partial {G_{xx}}/{\partial (\phi /\phi _0)}$ from panel (**a**).

An alternative approach to reveal the quantization effect through sub-band formation is to apply an in-plane magnetic field. In the presence of a magnetic field applied along the TI nanoribbon, Eq. 3 can be rewritten as:

6 $$\begin{aligned} E_l(k) =\pm \hbar v_F \sqrt{k^2+\frac{\pi (l-\phi /\phi _0)^2}{S}}, \end{aligned}$$

where $\phi =BS$ is a magnetic flux threading the cross-section area *S* of the nanoribbon, and, $\phi _0=h/e$ is the fundamental flux quantum. According to Eq. 6, the energy-momentum relation is periodic in $\phi /\phi _0$, which leads to the appearance of AB oscillations of $G_{xx}$^47^ (Fig. 8a).

In the quasi-ballistic regime, *h*/*e* AB oscillations are predicted to dominate over *h*/2*e* AAS oscillations^47^. AAS oscillations originate from interference between time-reversed paths, due to weak anti-localization and, thus, differ from AB oscillations in their physical origin. In this regime, the circumference of the nanoribbon should not be much longer than the mean-free path $l_e$. The pattern of these oscillations also depends on the carrier density, which can be changed by applying a gate voltage. At the Dirac point, magnetoconductance minimum at $\phi /\phi _0 = 0$ and maximum at $\phi /\phi _0=0.5$ should be observed. However, even away from the Dirac point, the AB phase can alternate with applied gate voltage, when $E_F$ is crossing the sub-bands^47^. Fig. 8 presents the magnetoconductance as a function of two different back-gate voltages (0 and $-8$ V), demonstrating that maxima and minima at finite magnetic flux alternate with an applied gate and the period oscillation at $V_g=0$ V is close to $\phi _0$ as shown by the Fourier transform of $\partial {G_{xx}}/{\partial (\phi /\phi _0)}$.

However, there is also a component in the FT at approximately twice the frequency that can be attributed to AAS. This can be explained by the presence of a strong weak anti-localization peak (Fig. 6a), which indicates that a fraction of modes has a more diffusive character. Since the gate voltage was not high enough to tune the chemical potential to the Dirac point, the alternating maxima and minima of $G_{xx}$ as a function of gate voltage indicate the crossing of sub-bands by the Fermi level. This behavior has not previously been observed in non-topological systems^47^. The fact that by decreasing the gate voltage the ratio between the AB and AAS inverts (Fig. 8b) might be due to more diffusive modes contributing at $V_g=-8$ V.

## Conclusions

In summary, we have demonstrated the reproducible and controllable growth of large quantities of ultrathin 3D-TI ${\mathbf {{{Bi}_{2}}}\mathbf {{Se}_{3}}}$ nanoribbons via physical vapour deposition, along with their systematic transport characterisation. The resulting nanoribbons exhibit Hall mobility in the range of 1000–2000 cm^2^/V·s, often up to an order of magnitude higher than in many ternary or quaternary topological insulator compounds, such as (Bi$_{1-x}$Sb$_x$)_2_Te_3_ or Bi$_{2-x}$Sb$_x$Te$_{3-y}$Se$_y$^17,50–53^.

We show that the nanoribbon growth kinetics can be tuned from a 2D layer-by-layer to a rough mode by adjusting synthesis parameters. A strong correlation between nanoribbon morphology and transport properties is demonstrated. The oscillatory pattern of magnetoresistance observed over the entire range of the magnetic field for ultrathin nanoribbons is explained by coherent Altshuler-Aronov-Spivak-like orbits with similar characteristic areas, which become accessible due to the specific surface morphology. Additionally, the superposition effect between AAS and SdH oscillations is observed at high magnetic fields.

While the main achievement of this study is the demonstration of a scalable and cost-effective route to ultrathin nanoribbons, we have *occasionally* obtained several ultranarrow nanoribbons with widths below 100 nm and thicknesses under 15 nm. In these exceptional cases, the nanoribbon circumference becomes smaller than the dephasing length, making sub-band quantization effects (manifesting as an oscillatory behaviour of the magnetoresistance as a function of back-gate voltage) significant in transport measurements.

The proposed growth method allows effective control over thickness via deposition time; however, the width of the nanoribbons remains dependent on the lateral dimensions of the seed plates. One potential approach to overcome this limitation is combining the time-controlled growth parameters demonstrated in this study with a Vapor-Liquid-Solid (VLS) approach, using catalyst particles to define ribbon width. This hybrid strategy could enable systematic growth of ultranarrow and ultrathin nanoribbons. It can be developed based on our previous experience with VLS growth of ${\mathbf {{Bi}_{2}}\mathbf {{Se}_{3}}}$ (used for high-yield growth of thicker nanoribbons^54^), and provides a promising route to achieving surface-state-dominated transport in highly confined topological insulator nanostructures.

## Supplementary Information

Supplementary Information.

## Data Availability

Data sets generated during the current study are available from the corresponding author on reasonable request.
