# Supplementary material for: Quantum confinement and coherent transport in ultrathin [image] nanoribbons
Source: Sci Rep. 2025 Oct 31;15:38272. doi: 10.1038/s41598-025-23622-7 (PMC12578795; doi:10.1038/s41598-025-23622-7)
Supplement: Supplementary file 1 — Supplementary Information. [file 41598_2025_23622_MOESM1_ESM.pdf]

Quantum Confinement and Coherent Transport in Ultrathin Bi_2Se_3 Nanoribbons

Kiryl Niherysh[1,2], Xavier Palermo[1], Ananthu P. Surendran[1], Alexei Kalaboukhov[1], Raitis Sondors[2], Jana Andzane[2], Donats Erts[2], Thilo Bauch[1], and Floriana Lombardi[1,*]

[1]Quantum Device Physics Laboratory, Department of Microtechnology and Nanoscience, Chalmers University of Technology, Göteborg, SE-41296, Sweden
[2]Institute of Chemical Physics, Faculty of Science and Technology, University of Latvia, Riga, LV-1586, Latvia
[*]floriana.lombardi@chalmers.se

SUPPLEMENTARY INFORMATION 1

Background subtraction

To obtain more detailed information from the SdH oscillations, the oscillatory component of the longitudinal resistance was extracted using the background subtraction method. Several background curves were obtained by interpolating the raw data with polynomials of different orders. As can be seen from Fig. S1a, the use of quadratic polynomial subtraction does not completely eliminate the background magnetoresistance, which prevents a clear determination of the SdH frequency. However, subtracting a higher order polynomial (for example, 5th order, Fig. S1b) more accurately defines peaks of the SdH oscillations from the raw data (see inset of Fig. 5a in the main text).

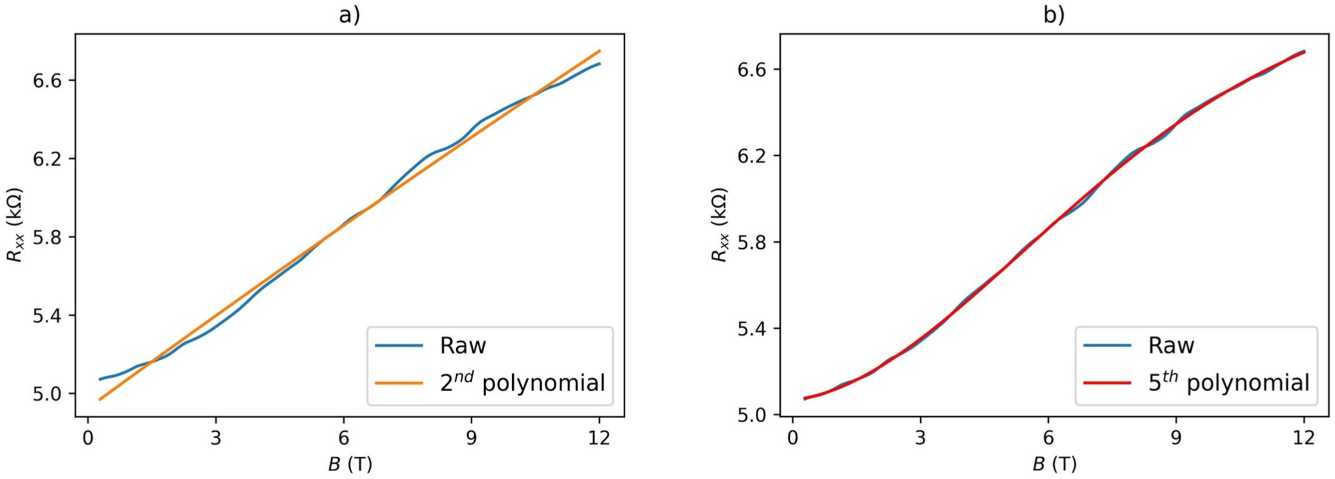

Figure S1. Subtraction of R_{xx} background (**Device II**) with a) 2nd and b) 5th order polynomial, respectively.

SUPPLEMENTARY INFORMATION 2

UCF analysis

Due to the specific surface morphology of our thin nanoribbons, unusually strong periodic conductance oscillations were observed across the entire range of magnetic fields. Here, we performed a more deep analysis of the unconventional behaviour of UCF, to show that these periodic oscillations originate from Altshuler-Aronov-Spivak (AAS)-type orbits with a similar characteristic area, existing due to the peculiar morphology of the nanoribbons.

Since the conductance G of each sample varies from each other, the root mean square (RMS) of the statistical distribution of conductance fluctuations ΔG_{RMS}=$\sqrt{\langle(\Delta G^2)\rangle} \approx e^2/h$ with $\Delta G = G - \langle G\rangle$, where $\langle G\rangle$ represents the ensemble averaging, is independent of material, size and degree of disorder and called Universal Conductance Fluctuation (UCF)[1].

Fig. S2a represents ΔG data measured for **Device I**. It is well known that UCF amplitudes decrease on average when the sample dimensions are longer than dephasing length L_φ [1,2], for example UCF in range from 6 to 0.01 e^2/h have been reported in Bi_2Se_3[3]. The values of ΔG^{I}_{RMS} = 0.049 and ΔG^{III}_{RMS} = 0.01 e^2/h were calculated for **Device I** and **Device III**, respectively. Considering simple 1D mesoscopic nanoribbon[1] (where only the length of the device is longer than L_φ) for our device's geometries we find that the expected ΔG_{RMS} ranges between 0.015 and 0.03 e^2/h (for L_φ of the order of 300 nm (see *Ref.*[4]), which is comparable with what we calculated above.

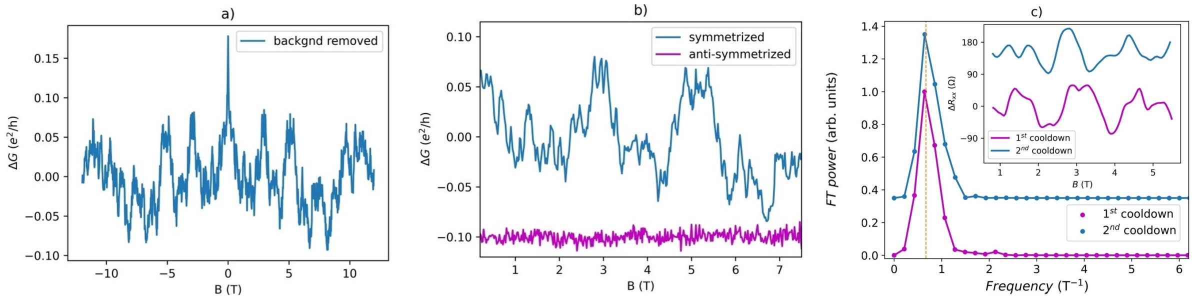

Figure S2. a) The conductance fluctuations $\Delta G = G - \langle G \rangle$ after subtraction of polynomial background as a function of magnetic field (**Device I**). b) Splitting the conductance fluctuations from panel a) into the symmetrical $\Delta G_s(B)$ and anti-symmetrical $\Delta G_a(B)$ components. To show the significant difference, the anti-symmetrized curve is shifted by -0.1 e^2/h. c) The Fourier transform of the conductance fluctuations (**Device III**) after subtraction of polynomial background as a function of a magnetic field (shown in the inset) measured after first (purple) and second (blue) cooldowns. The measurements were carried out at a temperature of 2 K. The dashed orange line represents frequency $F^{III}_{UCF\_1CD} \approx F^{III}_{UCF\_2CD} = 0.72$ T^{-1}.

As can be seen from Fig. 5b (in the main text), **Device I** with the exception of the dominant frequency F^{I}_{UCF} = 0.41 T^{-1} attributed to AAS-type orbits also demonstrates faster oscillations. To investigate the origin of these oscillations, the following analysis was carried out. The contribution to the conductance $\Delta G(B) = \Delta G_{int}(B) + \Delta G_n(B)$ consists of static fluctuations, associated with interference (UCF) $\Delta G_{int}(B)$, and noise components $\Delta G_n(B)$, which are determined by the measuring equipment and the sample. To illustrate the effect of noise on the measured fluctuations, the fluctuations remaining after subtracting the polynomial background (Fig. S2a) have been converted to $\Delta G(B) = \Delta G_s(B) + \Delta G_a(B)$, where $\Delta G_s(B)$ is symmetrized and $\Delta G_a(B)$ is anti-symmetrized components. In its turn, the symmetric part is $\Delta G_s(B) = 1/2(\Delta G(B) + \Delta G(-B))$ and the anti-symmetric part is $\Delta G_a(B) = 1/2(\Delta G(B) - \Delta G(-B))$, respectively. Since interference-related conductance fluctuations must be symmetrical in the magnetic field[5], $\Delta G_{int}(B)$ can only occur in the symmetrical part of $\Delta G_s(B)$, while the noise component $\Delta G_n(B)$ will be present equally in the symmetric $\Delta G_s(B)$ and anti-symmetric $\Delta G_a(B)$ components. Therefore $\Delta G_s(B) = \Delta G_{int}(B) + \Delta G^s_n(B)$, and $\Delta G_a(B) = \Delta G^a_n(B)$[6]. In Fig. S2b the conductance fluctuations $\Delta G = G - \langle G \rangle$ measured for **Device I** are presented as a function of a magnetic field $\Delta G(B)$ converted to symmetric (UCF+noise) and anti-symmetric (noise) components. It is clearly seen, that after splitting the noise component, device still experiences huge oscillations with period ≈ 2.5 T (which we classified as AAS-type oscillations) and the conventional aperiodic UCF.

Another possible mechanism explaining B-periodic oscillations of transport modes is the Sondheimer size effect[7,8]. However, Sondheimer oscillations (SO) should influence both longitudinal and transverse magnetotransport channels. Due to the significantly larger value of R_{xx} than the Hall resistance R_{xy} the SO should be more clearly visible against the background during Hall measurements, and should also be present in the longitudinal channel[7]. Nevertheless, in our particular case (**Device I**), the measured Hall effect is linear, while pronounced oscillations are observed only in the $R_{xx}(B)$ dependence (Fig. 4). Although derived to describe surfaces, the Fuchs-Sondheimer theory[9,10] does not consider the influence of surface states and quantum size effects in the case of topological insulators[11,12]. All the above-mentioned facts allow us to exclude the SO mechanism from the explanation of the transport properties of thin TI nanoribbons.

Since we associate periodic UCF with the peculiar morphology of the nanoribbon surface, which may differ from sample to sample, we specified the UCF frequency for each measured device. For instance, $F^{I}_{UCF} = 0.41$ T^{-1} for **Device I** and $F^{III}_{UCF} = 0.72$ T^{-1} for **Device III**, respectively. We also checked the change in UCF frequency after the first and second cooldowns for **Device III**. Despite the differences in the oscillation patterns (Fig. S2c (inset)), the FT of the oscillations in a magnetic field below 5.5 T shows the same frequency in both cooldowns: $F^{III}_{UCF-1CD} \approx F^{III}_{UCF-2CD}$, each approximately equal to 0.72 T^{-1}. Moreover, a shift of the above-mentioned frequency following $1/\cos\theta$ dependence, when angle θ increases from 0 up to 30 deg ($F^{III}_{UCF-0deg} = 0.72$ and $F^{III}_{UCF-30deg} = 0.83$ T^{-1}) was observed. This fact together with a clear alignment (superposition) of magnetoresistance fluctuations associated with the UCF as a function of the perpendicular component of the magnetic field $B_\perp = B\cos\theta$ (Fig. 6b) indicate the 2D nature of the UCF[13,14] and consistent with the hypothesis about Altshuler-Aronov-Spivak like orbits associated with the morphology of nanoribbons.

SUPPLEMENTARY INFORMATION 3

Size determination of AAS orbits

To determine the averaged size of AAS orbits an additional high-resolution AFM measurement was performed for a nanoribbon from **Device I** (Figs. S3a,b). To calculate the characteristic size of the irregularities, the surface morphology was analyzed using the two-dimensional fast Fourier transform (2D-FFT) technique.

Fig. S3c shows the frequency spectrum image. In the case of isotropic material, the power spectrum is radially symmetric, and a radial distribution can be distinguished. From the averaged distribution of the power spectrum intensity, one can extract the averaged frequency and, therefore, the averaged diameter of the orbits. From the profile shown in Fig. S3d, we determined the dominant frequency of 0.034±0.002 nm^{-1} using a Gaussian fit. This frequency corresponds to a characteristic length scale in the real space of the original image[15]. The extracted averaged size (diameter) of possible orbits L = 29.4±1.6 nm is in good agreement with a characteristic diameter of AAS orbits ($2R_{AAS}$ = 32 nm), extracted from the transport measurements.

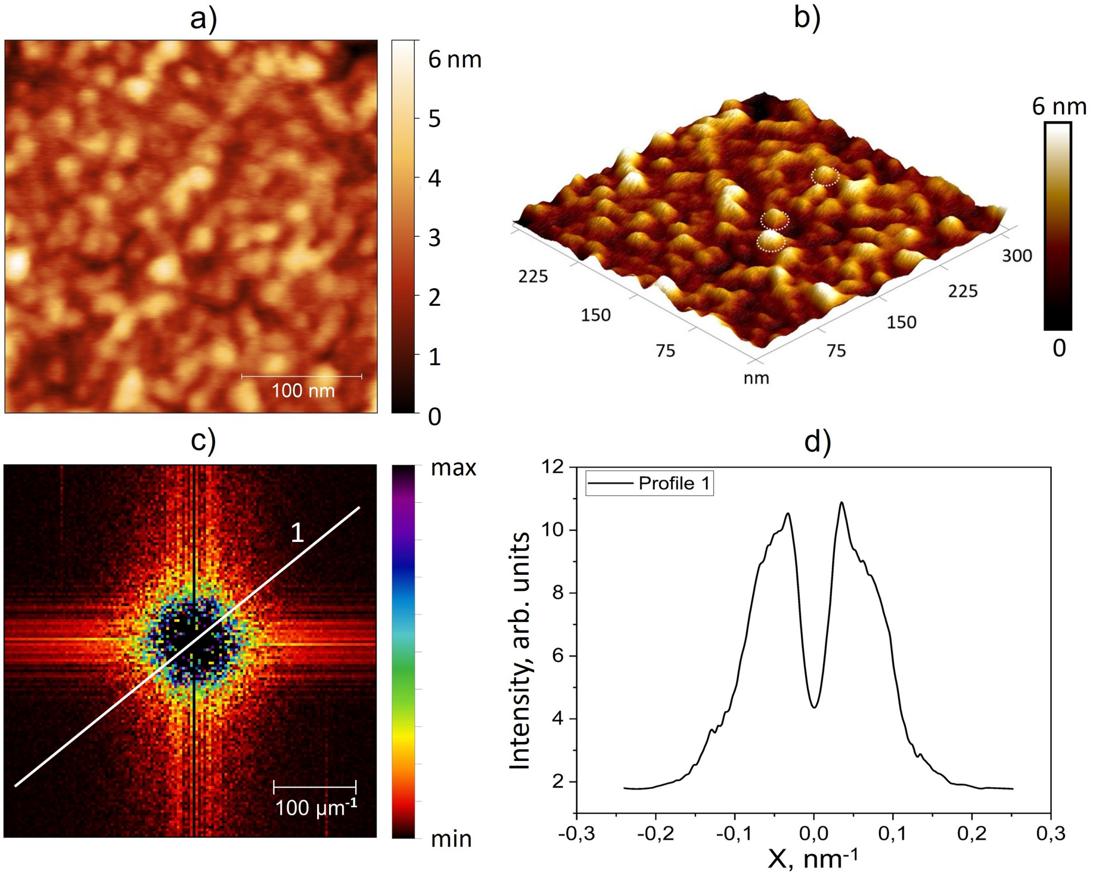

Figure S3. AFM measurements of the nanoribbon surface (**Device I**). a) Scan size of 310 × 310 nm^2. b) 3D AFM plot of image presented in panel a). White dashed circles delimitate possible AAS orbits. c) The frequency spectrum image of data from panel a). d) Profile 1 taken from 2D FFT-map from panel c): the frequency value at the peak corresponds to 0.034±0.002 nm^{-1}.

SUPPLEMENTARY INFORMATION 4

Feasibility of AAS Oscillations

Altshuler-Aronov-Spivak (AAS) oscillations in magnetoconductance, arising from quantum interference, have been observed in numerous experiments and are known to occur in both (quasi)-ballistic and diffusive transport regimes[16]. And the characteristic length scale over which such quantum interference can occur is the phase coherence length (if the orbit length is comparable to or smaller than L_ϕ)[17]. To compare the characteristic length of AAS orbits with the phase coherence length, we performed a weak anti-localization (WAL) analysis on nanoribbons from **Device I** (w = 360 nm, t = 12 nm) using the two-dimensional localization theory based on the Hikami-Larkin-Nagaoka (HLN) equation[18] (see Fig. S4a).

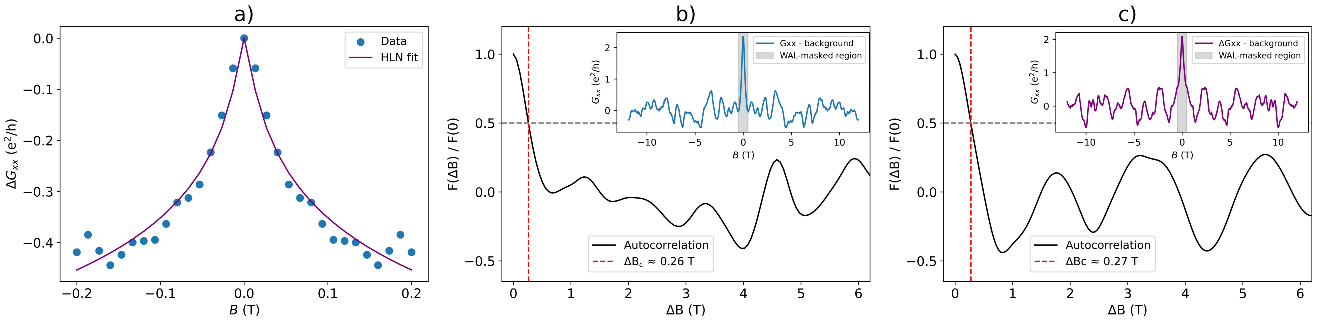

Figure S4. a) Weak anti-localisation (WAL) peak fitted using the HLN equation, yielding a phase coherence length $L_\phi \approx 320$ nm (**Device I**). The fitted prefactor $\alpha = -0.5$ indicates strong spin-orbit coupling and suggests transport dominated by a single coherent conduction channel, consistent with topological surface states. b-c) Autocorrelation function $F(\Delta B) = \langle \Delta G(B) \Delta G(B + \Delta B) \rangle$ of the conductance fluctuations for **Device III**, calculated after subtracting a 5th-order polynomial background and removing the WAL cusp. Data are shown for the second cooldown b) and the first cooldown c). The vertical red dashed line marks the correlation field ΔB_c, defined by the condition $F(\Delta B_c) = F(0)/2$ (indicated by the horizontal grey dashed line). The correlation field is related to the phase coherence length via $B_c = 0.95(h/e)(1/L_\phi \times w)$, where w is the nanoribbon width.

The extracted $L_\phi \approx 320$ nm. The fitted prefactor $\alpha = -0.5$ indicates strong spin-orbit coupling[18,19] as well as the presence of a single coherent transport channel (surface states)[20]. However, for **Device III**, where the ribbon width $w = 85$ nm, which is smaller than the typical $L_\phi \approx 300$ nm for Bi_2Se_3[4,21], the HLN model is not strictly applicable in its standard 2D form. Instead, we estimated L_ϕ from the analysis of universal conductance fluctuations (UCF), using the autocorrelation function of the conductance after subtracting a polynomial background and removing the WAL peak (Figs. S4b and c). The resulting value of L_ϕ for this device is ≈ 180 nm. Also, for **Device III**, we evaluated the size of the AAS orbits in the same manner as described in the main text for **Device I**. Using $F^{III}_{UCF} = 0.72\ \mathrm{T}^{-1}$, the corresponding AAS orbit length is $L^{III}_{AAS} = 137$ nm, yielding an effective loop radius of approximately 22 nm. As shown for both devices, the calculated AAS orbit length is smaller than the corresponding phase coherence length.

For **Device I** (which contains a Hall bar geometry), we estimate the electron mean free path using the relation $l_e = \frac{\hbar\mu}{e}\sqrt{2\pi n_{2D}}$. With a mobility $\mu = 1075\ \mathrm{cm^2/Vs}$ and a 2D carrier density $n_{2D} = 1.21 \times 10^{13}\ \mathrm{cm^{-2}}$, we obtain $l_e \approx 62$ nm, suggesting that the observed AAS occur in the diffusive transport regime.

SUPPLEMENTARY INFORMATION 5

Effect of Thermal Cycling on Magnetotransport Reproducibility

The magnetotransport in the fabricated nanoribbons exhibits complex behaviuor, including universal conductance fluctuations (UCF), Altshuler-Aronov-Spivak (AAS) and Shubnikov-de Haas (SdH) oscillations. These phenomena are often superimposed and difficult to disentangle in practice. The variation in magnetoresistance observed after thermal cycling can be attributed to the sensitivity of UCF to microscopic changes in the disorder potential, which are known to occur after warming to room temperature. In the Fig. S5 we present magnetoconductance measurements in the range of ± 1 T, recorded after the second cooldown for **Device III**, as a function of temperature from 2 to 20 K. It can be seen that during the same cooldown, the phase of the magnetoconductance oscillations remains stable across the entire temperature range. This observation supports the assumption, that the phase shifts observed between different cooldowns arise from changes in the disorder configuration, rather than from thermal effects during a single cooldown.

SUPPLEMENTARY INFORMATION 6

Reproducibility of Gate-Dependent Sub-Bands Oscillations

In addition, here we present gate voltage-dependent measurements recorded after a second cooldown. Several sweeps were performed with varying voltage step sizes (including fine steps of 10 mV to ensure high resolution). While the overall magnetoresistance background changed between cooldowns, the gate-dependent oscillations remained highly consistent in both frequency and amplitude across all sweeps (Fig. S6). These oscillations correspond to the expected energy scales for

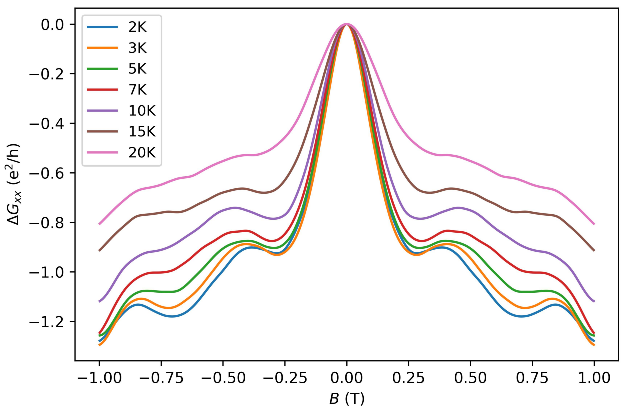

Figure S5. Magnetoconductance of **Device III** measured as a function of magnetic field at different temperatures (2-20 K), after the second cooldown. The phase of the oscillations remains unchanged with temperature, indicating phase stability during a single cooldown.

sub-band quantization, as discussed in detail in the main manuscript. This strongly supports the interpretation that the observed oscillations arise from well-defined sub-bands rather than disorder effects.

In total, six independent sweeps were conducted under different conditions (two cooldown cycles and multiple sweep rates). In every case, the oscillations were reproducible and stable without the need for averaging. These results strongly support the presence of sub-band quantization and effectively rule out alternative explanations.

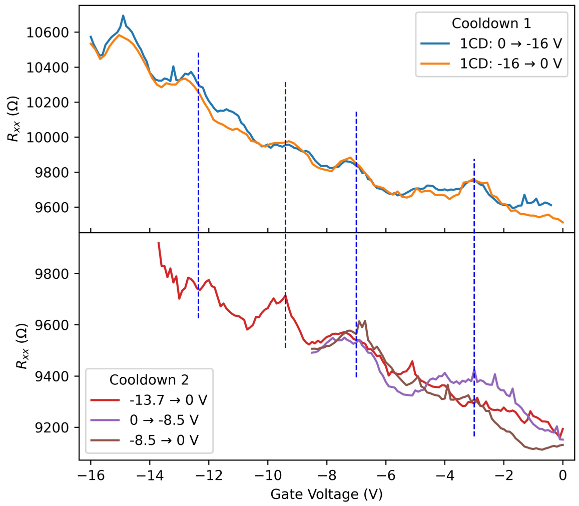

Figure S6. R_{xx} of **Device III** as a function of back-gate voltage. The top panel shows measurements performed after the first cooldown (as presented in the main manuscript), with different colors representing opposite sweep directions. The bottom panel displays additional gate sweeps taken after a second cooldown, performed with varying gate voltage step sizes. Dashed vertical lines indicate oscillation peaks occurring at the same gate voltages across both cooldowns, highlighting the stability of the sub-band features.

SUPPLEMENTARY INFORMATION 7

Bottom Surface Dominated Gate Response

In the main manuscript, we present a schematic model of charge carrier concentration as a function of Fermi energy (Fig. 7b), where the blue line represents the contributions of the top and bottom Dirac surface states to the total carrier density. For **Device III**, we assume that carrier modulation within the applied gate voltage range occurs primarily at the bottom surface states.

This assumption is supported by previous work from our group[4], in which a two-carrier model was used to analyze longitudinal and transverse magnetoconductance as a function of gate voltage. This analysis allowed the extraction of carrier concentrations for the different conduction channels. One channel corresponds to Dirac electrons at the top surface, while the second channel combines the 2D accumulation layer at the substrate/nanoribbon interface and the Dirac electrons at the bottom surface of the nanoribbon. The bulk contribution was neglected due to the ultrathin nature of the nanoribbons.

The extracted gate-dependent carrier densities show that the second channel (bottom surface + interface) decreases sharply with changes in back-gate voltage, while the first channel (top surface) initially contributes only a minor fraction. After the Dirac point is crossed (at $V_g \approx -10$ V), the second channel is nearly depleted, resulting in stronger modulation of the top surface carrier density.

We note that the substrate used in *Ref.*[4] differs from that in the present study, featuring a higher dielectric constant. This allows more efficient carrier depletion and easier access to the Dirac point. Therefore, in our **Device III**, we assume that the primary carrier modulation occurs in the bottom surface states within the applied back-gate voltage range.

References

1. Lee, P. A., Stone, A. D. & Fukuyama, H. Universal conductance fluctuations in metals: Effects of finite temperature, interactions, and magnetic field. *Phys. Rev. B* **35**, DOI: https://doi.org/10.1103/PhysRevB.35.1039 (1987).
2. Checkelsky, J. G. *et al.* Quantum Interference in Macroscopic Crystals of Nonmetallic Bi_2Se_3. *Phys. Rev. Lett.* **103**, DOI: https://doi.org/10.1103/PhysRevLett.103.246601 (2009).
3. Li, Z. *et al.* Two-dimensional universal conductance fluctuations and the electron-phonon interaction of surface states in Bi_2Te_2Se microflakes. *Sci. Reports* **2**, DOI: https://doi.org/10.1038/srep00595 (2012).
4. Kunakova, G. *et al.* High-mobility ambipolar magnetotransport in topological insulator Bi_2Se_3 nanoribbons. *Phys. Rev. Appl.* **16**, DOI: https://doi.org/10.1103/PhysRevApplied.16.024038 (2021).
5. Washburn, S. & Webb, R. A. Aharonov-Bohm effect in normal metal quantum coherence and transport. *Adv. Phys.* **35**, DOI: https://doi.org/10.1080/00018738600101921 (1986).
6. Bauch, T. *Quanteninterferenzeffekte und zeitabhängiger elektronischer Transport in metallischen Nanostrukturen*. Ph.D. thesis, Universität zu Köln. URL: https://kups.ub.uni-koeln.de/475 (2000).
7. van Delft, M. R. *et al.* Sondheimer oscillations as a probe of non-ohmic flow in WP_2 crystals. *Nat. Commun.* **12**, DOI: https://doi.org/10.1038/s41467-021-25037-0 (2021).
8. Mallik, S. *et al.* From Low-Field Sondheimer Oscillations to High-Field Very Large and Linear Magnetoresistance in a $SrTiO_3$-Based Two-Dimensional Electron Gas. *Nano Lett.* **22**, DOI: https://doi.org/10.1021/acs.nanolett.1c03198 (2022).
9. Sondheimer, E. H. The Influence of a Transverse Magnetic Field on the Conductivity of Thin Metallic Films. *Phys. Rev.* **80**, DOI: https://doi.org/10.1103/PhysRev.80.401 (1950).
10. Fuchs, K. & Wills, H. H. The conductivity of thin metallic films according to the electron theory of metals. *Math. Proc. Camb. Philos. Soc.* **34**, DOI: https://doi.org/10.1017/S0305004100019952 (1938).
11. Hinsche, N. F. *et al.* Impact of the topological surface state on the thermoelectric transport in Sb_2Te_3 thin films. *ACS Nano* **9**, DOI: https://doi.org/10.1021/acsnano.5b00896 (2015).
12. Tang, M. *et al.* Ultrathin Topological Insulator Absorber: Unique Dielectric Behavior of Bi_2Te_3 Nanosheets Based on Conducting Surface States. *ACS Appl. Mater. Interfaces* **11**, DOI: https://doi.org/10.1021/acsami.9b13775 (2019).
13. Lee, J., Park, J., Lee, J.-H., Kim, J. S. & Lee, H.-J. Gate-tuned differentiation of surface-conducting states in $Bi_{1.5}Sb_{0.5}Te_{1.7}Se_{1.3}$ topological-insulator thin crystals. *Phys. Rev. B* **86**, DOI: https://doi.org/10.1103/PhysRevB.86.245321 (2012).
14. Kandala, A., Richardella, A., Zhang, D., Flanagan, T. C. & Samarth, N. Surface-sensitive two-dimensional magneto-fingerprint in mesoscopic Bi_2Se_3 channels. *Nano Lett.* **13**, DOI: https://doi.org/10.1021/nl4012358 (2013).

15. Carmona, P. *et al.* Structure evolution during phase separation in spin-coated ethylcellulose/hydroxypropylcellulose films. *Soft Matter* **17**, DOI: http://doi.org/10.1039/D1SM00044F (2021).

16. Basarić, F. *et al.* Aharonov-Bohm and Altshuler-Aronov-Spivak oscillations in the quasiballistic regime in phase-pure GaAs/InAs core/shell nanowires. *Phys. Rev. B* **112**, DOI: https://doi.org/10.1103/xljl-s1lp (2025).

17. Ferrier, M. *et al.* Direct Measurementof the Phase-Coherence Length in a GaAs/GaAlAs SquareNetwork. *Phys. Rev. Lett.* **93**, DOI: https://doi.org/10.1103/PhysRevLett.93.246804 (2004).

18. Hikami, S., Larkin, A. I. & Nagaoka, Y. Spin-Orbit Interaction and Magnetoresistance in the Two Dimensional Random System. *Prog. Theor. Phys.* **63**, DOI: https://doi.org/10.1143/PTP.63.707 (1980).

19. Matsuo, S. *et al.* Weak antilocalization and conductance fluctuation in a submicrometer-sized wire of epitaxial Bi_2Se_3. *Phys. Rev. B* **85**, DOI: https://doi.org/10.1103/PhysRevB.85.075440 (2012).

20. Akiyama, R., Fujisawa, K., Yamaguchi, T., Ishikawa, R. & Kuroda, S. Two-dimensional quantum transport of multivalley (111) surface state in topological crystalline insulator SnTe thin films. *Nano Res.* **9** (2016). DOI: https://doi.org/10.1007/s12274-015-0930-8 (2016).

21. Cha, J. J. *et al.* Effects of Magnetic Doping on Weak Antilocalization in Narrow Bi_2Se_3 Nanoribbons. *Nano Lett.* **12**, DOI: https://doi.org/10.1021/nl3021472 (2012).
